# Folic Acid Supplementation and Preterm Birth: Results from Observational Studies

**DOI:** 10.1155/2014/481914

**Published:** 2014-03-03

**Authors:** Elena Mantovani, Francesca Filippini, Renata Bortolus, Massimo Franchi

**Affiliations:** ^1^Sezione di Ostetricia e Ginecologia, Dipartimento di Scienze della Vita e della Riproduzione, Università degli Studi di Verona, Piazzale Ludovico Scuro 10, 37134 Verona, Italy; ^2^Azienda Ospedaliera Universitaria Integrata Verona, Piazzale Stefani 1, 37126 Verona, Italy

## Abstract

*Introduction*. Folic acid (FA) supplementation is recommended worldwide in the periconceptional period for the prevention of neural tube defects. Due to its involvement in a number of cellular processes, its role in other pregnancy outcomes such as miscarriage, recurrent miscarriage, low birth weight, preterm birth (PTB), preeclampsia, abruptio placentae, and stillbirth has been investigated. PTB is a leading cause of perinatal mortality and morbidity; therefore its association with FA supplementation is of major interest. The analysis of a small number of randomized clinical trials (RCTs) has not found a beneficial role of FA in reducing the rate of PTBs. *Aim of the Study*. The aim of this review was to examine the results from recent observational studies about the effect of FA supplementation on PTB. *Materials and Methods*. We carried out a search on Medline and by manual search of the observational studies from 2009 onwards that analyzed the rate of PTB in patients who received supplementation with FA before and/or throughout pregnancy. *Results*. The results from recent observational studies suggest a slight reduction of PTBs that is not consistent with the results from RCTs. Further research is needed to better understand the role of FA supplementation before and during pregnancy in PTB.

## 1. Introduction

Folates are a group of water-soluble B vitamins naturally existing in food such as legumes, leafy green vegetables, and some fruits.

Folate in foods is naturally present in the form of reduced folate polyglutamate conjugates, while folic acid (FA) refers to the fully oxidized and most stable form of folate [1]. Due to a better bioavailability of FA, which is approximately 70% higher than that of folate, this is the synthetic form often used in supplements and in fortified foods [2].

Under fasting conditions, the bioavailability of FA approaches 100% reducing to about 85% when consumed with food [3], while for food folates it is estimated to be around 50% (60–90% for some fruits and vegetables) [4].

This group of vitamins acts as substrates and cofactors in many cellular pathways and biological reactions like DNA and RNA synthesis, cell replication, intracellular signalling, and gene programming through methylation, among the others.

All these processes are implicated in fetal and placental growth and development; therefore the embryonic tissues are sensitive to the presence of folates deficit or metabolic alterations [5, 6].

In particular, methylation is the target of recent researches due to the suggested role of folates in epigenetic changes that would modify the individual response to diseases later in life [7].

Supplementation with FA would therefore lead to different and more widespread effects during specific critical developmental windows in pregnancy, as demethylation and subsequent remethylation occur in the early embryo [8].

Recommended dietary allowances for folate, expressed as dietary folate equivalents (DFE)—which takes into account the higher bioavailability of synthetic FA compared to naturally occurring folate in food—are 400 *μ*g DFE/day in males and females over 19 y and 600 *μ*g DFE/day for pregnant women [3, 9].

Normal values (NV) of folates can be assessed in different ways. Red blood cells (RBC) folate concentration (NV > 316 nmol/L (140 ng/mL)) is defined as a primary indicator of folate status because of its correlation with liver folate and consequent tissue stores [10]. Other indicators of folate status are serum folate (NV > 6.8 nmol/L (3 ng/mL)) and homocysteine (NV < 14 *μ*mol/L) [6].

The pharmacokinetics of FA shows a linear increase in the steady-state concentration of serum folate levels for increasing dose, when supplemented doses are administered [11].

Wald and collaborators indicate that serum folate concentrations increase by 0.94 ng/mL (95% CI 0.77–1.10) for every 0.1 mg/day increase of FA intake in women aged 20–35 years [12]. In addition to that, the absorption of FA is similar whether it is administered in the morning or evening, showing no circadian variation [13].

In literature an estimated mean adherence rate of 55% among pregnant women taking prenatal multivitamins is reported [14]; thus a higher FA supplementation could better maintain the serum folate concentrations. For example, the estimated steady-state serum folate levels produced by 5 mg FA would remain above 7 ng/mL, even with 24% of pills taken [11].

However, the mean time to reach a RBC concentration of folate >906 nmol/L seems to be 4.2 ± 3.5 weeks even with a supplementation of 0.8 mg of FA [15]. In another study plasmatic levels of folates reached the plateau level after 3 months of supplementation with an increase in serum folate by 80% in the first month with all doses (100 *μ*g/die, 400 *μ*g/die, 4000 *μ*g/die, 4000 *μ*g/week) [16].

Despite the unequivocal success of FA in reducing neural tube defects (NTDs) rates [17–19], concerns have been raised about the potential adverse effects of exposure to unmetabolized FA resulting from FA intakes that overwhelm the liver metabolic capacity [20].

However, according to recent researches some compensatory mechanisms exist to limit this exposure: a high dose (5 mg) of FA roughly doubles the median plasma concentration of unmetabolized FA over the first 12 weeks of supplementation, but a significant decline is observed thereafter, despite ongoing supplementation and sustained total folate concentrations [21].

In addition to that, plasma concentration of unmetabolized FA does not appear significantly different between groups exposed to 1.1 mg and 5 mg, respectively [11].

For security concerns also unmetabolized FA concentration in cord blood has been investigated and it seems that, in the majority of pregnant women, FA supplied is not likely to accumulate in the fetus [20, 22].

In pregnancy an increase of folate breakdown products has been observed, in line with the extra demand due to the rapidly growing placenta and fetus [23, 24] suggesting a possible relation of folate status in the mother on fetal growth [25]. Also, folates are required for the metabolism of homocysteine, whose level is associated with pregnancy complications, such as miscarriage, placental abruption, and hypertension disorders [26–29].

A relevant scientific literature supports the role of FA in protecting against both the first occurrence [17, 18] and the recurrence [19, 30] of NTDs when used in the periconceptional period. For this reason expert committees worldwide have issued recommendations about supplementation with 0.4–1 mg of FA, or 4-5 mg if at higher risk of having a baby with NTD [19, 31–34], and some countries adopted a policy of food fortification with FA [35].

In the Cochrane review of De-Regil et al., the issue of different doses, forms and schemes of folate supplementation in the studies available is reported. In this review it is confirmed that periconceptional (started before conception and continued until 12 weeks of gestational age) FA supplementation prevents NTDs, but there were insufficient data to evaluate the effects on other outcomes such as congenital cardiovascular defects, cleft lip and palate, miscarriages, or any other birth defects [36].

Among the other outcomes, the rate of preterm birth (PTB), which indicates birth before 37 weeks of gestational age, has a major interest being a leading cause of perinatal morbidity and mortality with a frequency of about 12-13% births in the USA and 5–9% in many other developed countries. These premature births are both medically induced for maternal or fetal complications (such as preeclampsia or eclampsia and intrauterine growth restriction) and spontaneous [37].

The survival chances of the 15 million babies born preterm each year vary dramatically depending on where they are born. South Asia and sub-Saharan Africa account for half the world's births, more than 60% of the world's preterm babies and over 80% of the world's 1.1 million deaths due to preterm birth complications [38].

There are some hypotheses that could link reduced blood folate and prematurity.

First, periconception FA supplementation may influence early placentation processes [39]. In fact FA is potentially important in a number of crucial early stages of placental development, including extravillous trophoblast invasion, angiogenesis, and secretion of matrix metalloproteinases [40].

The role of FA in placentation could be supported also by the observation that increased homocysteine levels (on which folates have a lowering effect [41, 42]) induce cytotoxic and oxidative stress on placental vascular and endothelial functions [43–45] and exposure of trophoblast cells to homocysteine may increase apoptosis [46]. Periconceptional supplementation may therefore be beneficial in preventing pregnancy disorders associated with deficient placental development that could lead to PTB [47–51].

Second, micronutrient status at the time of implantation could have a role in inflammation. In fact early PTB is often caused by intrauterine infection and according to some studies FA is reduced in PTB [52, 53].

However, the serum levels of homocysteine or FA measured in patients with preterm premature rupture of membrane, an important cause of preterm delivery, do not differ from those in matched control women [54, 55].

At last, recent findings have also shown an association between polymorphisms in folate metabolizing genes and the risk of spontaneous PTB [56].

The impact of PTB justifies the ongoing research on all possible preventive methods and, on this basis, the effects of supplementation with FA on prematurity have been recently investigated.

The Cochrane review of Lassi and collaborators [57] had the aim to assess the effect of FA supplementation alone taken during pregnancy on haematological and biochemical parameters and adverse events. Only 3 out of the 31 trials included considered PTB among the outcomes, though with different definitions (birth before 38 weeks of gestational age in 2 trials [58, 59]), and found that FA supplementation (from 200 mcg to 5 mg) during pregnancy (started after conception and continued until 30 weeks [60], administered before 26 weeks [58], started at 24 weeks and continued to delivery [59]) had no impact on PTB (2,959 patients, RR 1.01, 95% CI 0.73–1.38).

Fekete et al. [61] investigated the effect of FA supplementation (from 0.25 mg to 5 mg) started in the second trimester and assessed the dose-response relationship between folate intake and birth weight, placental weight, and length of gestation. Data from 5 RCTs involving 380 participants were analyzed. These studies reported a period of supplementation of 12–24 weeks and folate intake both from supplements or fortified foods and natural food source, often compared to the use of iron alone. No beneficial effect of folate supplementation on length of gestation was found.

Chiaffarino et al. [62] reviewed both RCTs and observational studies to investigate the effects of FA supplementation ranging from 200 mcg to 5 mg on pregnancy outcomes. In the majority of RCTs reported in this review, FA supplementation was started after conception (ranging from the first consultation in pregnancy to the 30th week of gestational age). The 3 observational studies reported were heterogeneous as they related the effects of different situations (preconceptional supplementation, FA intake in pregnancy, and homocisteine level in pregnancy) to PTB. The conclusion was that FA supplementation in different periods of pregnancy had no impact on the reduction of PTB according to RCTs while the analysis of the observational studies showed a potential beneficial role of FA.

## 2. Aim of the Study

The aim of our review is to update the findings of recent observational studies that investigated the effect of FA supplementation on PTB with interest in differentiating the period of use (periconceptional and postconceptional) and when available the outcome of spontaneous PTB.

## 3. Materials and Method

Medline database was searched using the keywords “folic acid,” “preterm,” “preterm delivery,” “prematurity,” “supplementation,” and “periconceptional” from 2009 onwards.

Reviews were excluded from this analysis and also non-English written observational studies. Only studies focusing mainly on FA supplementation and in singleton, low risk pregnancies were included. Studies that evaluated the outcome of PTB according to folate or homocysteine concentration in blood or serum were also excluded.

Finally, 7 papers were included.

The term PTB always refers to births that occurred before 37 weeks of pregnancy.

The studies evaluated were heterogeneous for the use of FA (alone or with multivitamins), the dose of FA and the beginning and the period of supplementation.

The characteristics and the results of the studies are indicated in Table 1.

## 4. Results

Our review has found that there is not any agreement among the results of the studies taken into the analysis.

The study of Papadopoulou [63] suggests a decrease of PTB in women supplemented with 5 mg of FA in early-midpregnancy. This result is confirmed also in a large cohort (38,151 women) [64] that used a similar dose of FA in the periconceptional period, but not in the “Generation R study” [65].

The use of FA in the second and third trimesters appears to be more protective against PTB than in the preconceptional period in a large cohort of patients that received supplementation in a fortification policy country (US) [66]. Differently, Alwan et al. reported an increased risk of PTB in a population with a later beginning of supplementation, especially if extended beyond the first and second trimesters of pregnancy. The authors report that it cannot be ruled out that residual confounding may be contributing to this apparent association as there may be unmeasured confounders resulting in the apparent negative relationship between multivitamin supplement taking in the third trimester and PTB [67].

Regarding the dose, more than 5 mg do not seem to increase the beneficial effect observed [63], while the length of supplementation (more than 1 year preconception and until the end of the first trimester) reduced the rate of PTB before 32 weeks of gestational age in a cohort of 34,480 women even if in this study the supplementation could include multivitamins [68].

Catov et al. [69] agree with the beneficial effect of multivitamins with FA (but not of FA alone) only in a subgroup of patients (nonobese women), while in overweight patients this effect was not observed.

The start of supplementation is an important issue as administration of FA before or after conception could have different effects due to its action on different targets.

Among the studies reported, the ones of Bukowski, Catov, and Timmermans evaluate the effect of FA given before conception on PTB, while the others report its use after that. Apparently, there is not any strong difference in the outcome.

Last, some studies report the effect of FA on the total number of PTB. However, differentiation between PTB due to spontaneous birth rather than to active interventions for maternal or fetal complications is an issue of relevant importance.

In our analysis there are some limitations due to the differences among these studies in terms of dose of FA, pre- or postconceptional beginning and end of supplementation, use of multivitamins, length of supplementation, and gestational age at which the supplementation was started if postconceptional.

A strong limitation of observational studies design is the possibility that relevant confounders have not been accounted for. Thus it is conceivable that folate supplementation could be a marker of healthy behavior. Also, the use of questionnaires based on women self-report use of FA or multivitamins could lead to the collection of questionable data and it is associated with recall bias. Comorbidities in the studied populations are not precisely described as well.

Instead, RCTs are the most rigorous way of determining whether a cause-effect relation exists between treatment and outcome and for assessing the cost effectiveness of a treatment. They have several important features such as random allocation to intervention groups, patients, and trialists unaware of the treatment given until the end of the study, identical treatment for all intervention groups (except for the experimental treatment), analysis of patients within the group to which they are allocated, irrespective of whether they experience the intended intervention (intention to treat analysis), and finally the analysis is focused on estimating the size of the difference in predefined outcomes between intervention groups.

## 5. Conclusion

In conclusion, our analysis shows that the results from the observational studies evaluated are conflicting, although a trend of PTB reduction in subgroups with definite features has been noted. These results are not entirely consistent with the ones derived from previous RCTs.

The impact of PTB on perinatal morbidity and mortality and the uncertain effect of FA supplementation on this condition require the development of well-designed and adequate sample size RCTs on this topic which examine the effect of preconceptional and postconceptional supplementation and differentiate the outcome in spontaneous and iatrogenic PTB.

## Figures and Tables

**Table 1:** Dose, period, and effect of FA supplementation on PTB in recent observational studies.

Author Year Reference Type of study	Study period	*N* women	Supplementation		Supplementation period	Recruitment	Birth outcomes	PTB/ population	PTB OR (95% CI)
Papadopoulou et al. 2013 [63] Cohort study	2007-2008	1,279	No use		Early to midpregnancy	14.6 ± 3.2 wk (14–18)	PTB sPTB LBW SGA	26/157	Reference^a^
			FA ± iron	ED 5 mg				90/849	0.69 (0.44–0.99) sPTB 0.57 (0.33–0.99)
				6–15 mg				30/273	0.72 (0.41–1.25) sPTB 0.71 (0.37–1.34)

Shaw et al. 2011 [66] Controls of case-control study	1998–2005	5,912	MV with FA	Dose not reported	1 month before conception 1st trimester 2nd-3rd trimester Sporadic use No use, before or during pregnancy	6 wk after estimated delivery–24 months after delivery	PTB LBW	158/1,697	Reference^b^
								197/2612 29/380 62/826 28/293	0.69 (0.52–0.92) 0.54 (0.31–0.93) 0.70 (0.46–1.04) 0.86 (0.49–1.50)

Catov et al. 2011 [69] Cohort study	1997–2003	35,897	No use		4 wk before-8 wk after last menstrual period	11.1 ± 3.9 wk (5–24 wk)	PTB SGA	604/11,503	Reference^c^
			MV with FA	200 mcg				1,013/21,785	Any use, all women: 0.89 (0.80–0.99)
									Any use, BMI < 25: 0.84 (0.73–0.95)
									Any use, BMI ≥ 25: 1.03 (0.84–1.26)
			FA	200 mcg				137/2,609	1.00 (0.91–1.11)

Alwan et al. 2010 [67] Cohort study	2003–2006	1,234	FA/MV with FA	Dose not reported	1st trimester 2nd trimester 3rd trimester	8–12 wk	PTB BW SGA	1,340* 229 82	1.3 (0.6–2.7)^d^ 1.8 (0.8–4.1) 3.4 (1.2–9.6)

Czeizel et al. 2010 [64] Controls of case-control study	1980–1996	38,151	No use		II and III trimesters	after birth	PTB LBW	1,792/16,177 1,461/19,102 44/685 64/1,410	Reference^e^ 0.68 (0.63–0.73) 0.63 (0.46–0.86) 0.40 (0.31–0.51)
			FA	ED 5.6 mg					
			MV with FA	ED 0.85 mg					
			MV with FA + FA	ED 3.7 mg					

Bukowski et al. 2009 [68] Cohort study	1999–2002	34,480	No use		Preconception start	1st trimester	sPTB SGA PE Abruptio placentae	790/15,259	Reference^f^
			FA ± MV	Dose not	<1 Y			558/12,444	20–28 wk: 0.72 (0.40–1.28)
				reported					28–32 wk: 0.73 (0.47–1.13)
									32–37 wk: 0.93 (0.83–1.06)
					>1 Y			311/6,777	20–28 wk: 0.31 (0.11–0.90)
									28–32 wk: 0.53 (0.28–0.99)
									32–37 wk: 0.99 (0.85–1.15)

Timmermans et al. 2009 [65] Cohort study	2002–2006	6,353	No use			15.4 wk (10.2–24.8 wk)	PTB LBW SGA	1,877	Reference^g^
			FA	0.4–0.5 mg	Preconception start			2,493	0.88 (0.63–1.21)
					Start before 8 wk of gestational age			1,983 PTB not reported	0.75 (0.55–1.02)

Suppl.: supplementation; wk: weeks; ED: estimated dose; MV: multivitamin; sPTB: spontaneous preterm birth; PE: preeclampsia; BW: birth weight; LBW: low birth weight; SGA: small for gestational age. ^a^Adjusted for maternal age, education, Greek origin, prepregnancy BMI (kg/m^2^), smoking status, parity, and iron intake from supplements. ^b^Adjusted for other two sources of folate, infant birth weight, smoking, alcohol use, race/ethnicity, maternal education, and maternal age. ^c^Adjusted for maternal age, parity, BMI, smoking, and sociooccupational status. *Women taking FA/MV. 55 total PTB reported. ^d^Adjusted for salivary cotinine levels, self-reported alcohol intake, maternal age, maternal vegetarian diet, ethnicity, baby's sex, parity, Index of Multiple Deprivation score, educational attainment, past history of miscarriage, and long-term chronic illness in an unconditional logistic regression model. ^e^Adjusted for maternal age, socioeconomic status, and birth order. ^f^Adjusted for maternal age, body mass index, race and ethnicity, educational level, marital status, smoking, parity, and history of prior preterm birth and recruitment center. ^g^Adjusted for gestational age at birth (not prematurity), maternal age, height, weight, parity, ethnicity, fetal gender, educational level, and smoking.
